# Prevalence of Segmental Colitis Associated with Colonic Diverticulosis in a Prospective Cohort of Patients Who Underwent Colonoscopy in a Tertiary Center

**DOI:** 10.3390/jcm11030530

**Published:** 2022-01-20

**Authors:** Francesca Falangone, Gianluca Esposito, Stefano Angeletti, Emanuela Pilozzi, Vito Domenico Corleto, Emilio Di Giulio, Bruno Annibale, Marilia Carabotti

**Affiliations:** Medical-Surgical Department of Clinical Sciences and Translational Medicine, University Sapienza, 00189 Rome, Italy; francesca.falangone@uniroma1.it (F.F.); gianluca.esposito@uniroma1.it (G.E.); stefanoangeletti63@gmail.com (S.A.); emanuela.pilozzi@uniroma1.it (E.P.); vito.corleto@gmail.com (V.D.C.); emilio.digiulio@uniroma1.it (E.D.G.); marilia.carabotti@uniroma1.it (M.C.)

**Keywords:** colonic diverticulosis, histology, segmental colitis associated to diverticulosis, interdiverticular inflammation

## Abstract

In patients with colonic diverticulosis, the prevalence of segmental colitis associated with diverticulosis (SCAD) is debated. The aim of this study was to assess the prevalence of SCAD in consecutive patients with diverticulosis in a single tertiary center. Over a six-month period, consecutive adult patients with colonic diverticulosis were included. Patients with endoscopic signs of interdiverticular mucosal inflammation (erythema, friability, and ulcerations) were considered suspected SCAD and underwent multiple biopsy samplings to confirm diagnosis. Clinical features were collected from diverticulosis and suspected SCAD patients. In total, 367 (26.5%) of 1383 patients who underwent colonoscopy presented diverticulosis. Among diverticulosis patients, 4.3% (*n* = 16) presented macroscopic signs of interdiverticular mucosal inflammation and were identified as suspected SCAD. Compared to that of patients with diverticulosis, the age of suspected SCAD patients was significantly lower (60 ± 12.9 years (41.0–86.0) vs. 70 ± 10.6 years (38.0–93.0)) (*p* = 0.047). Among patients with suspected SCAD, one patient received a new diagnosis of Crohn’s disease, one had spirochetosis infection, and one presented drug-induced colitis. The remaining patients with suspected SCAD (*n* = 13) were not confirmed by histology. This observational study suggests that SCAD diagnosis is a challenge in clinical practice due to the heterogeneity of endoscopic findings and lack of stated histological criteria.

## 1. Introduction

Diverticulosis of the colon is common in Western countries, affecting 50–66% of individuals over 80 years of age [1]. A small but undefined subset of patients with diverticulosis may develop segmental colitis associated with diverticulosis (SCAD) [2].

SCAD is a clinical entity with macroscopic and microscopic features characterized by chronic mucosal inflammation involving the interdiverticular mucosa, typically the sigmoid-descending colon, and sparing the proximal and rectal colon [2]. Generally, males are more affected than females, with a median age of presentation of 63.6 years (range 26–87) [3]. The most common clinical manifestations are rectal bleeding, diarrhea, and abdominal pain. Weight loss, nausea, fever, and biochemical alterations such as leucocytosis are very rare [4,5,6]. The majority of these patients showed a benign course with spontaneous resolution or responded to 5-aminosalicylates [7,8].

To confirm SCAD diagnosis, correct biopsy sampling, including biopsies on the borders of the diverticula, in the colon proximal to the diverticular area, and in the rectum, is required [2]. The range of histological findings associated with SCAD is variable, including mild nonspecific inflammation and inflammatory bowel disease (IBD)-like changes, making differential diagnosis difficult [2,5].

The prevalence of SCAD in patients with diverticulosis is not yet defined, ranging from 1.9 to 11.4% [9,10]; it is likely that the different histological criteria and a lack of diagnostic standardized features contributed to this wide range of prevalence. In addition, the variability of biopsy samplings among available studies may further contribute to this heterogeneity. In fact, some authors based the diagnosis of SCAD mainly on endoscopic evidence of inflammation of the interdiverticular mucosa without performing adequate biopsy sampling (interdiverticular mucosa, rectum, and proximal colon), thus affecting the reported SCAD prevalence.

The aim of the present observational study was to assess the prevalence of SCAD in consecutive patients with colonic diverticulosis who underwent colonoscopy in a single tertiary center.

## 2. Materials and Methods

We prospectively enrolled consecutive adult patients who underwent colonoscopy for colorectal cancer screening (CCS) from 1 September 2019 to 28 February 2020, at a single tertiary university center or for other clinical diagnostic reasons, during which the presence of colonic diverticula was documented. Patient recruitment was stopped prematurely due to the COVID-19 health emergency, which interrupted routine endoscopic activity (study deadline expected on 30 August 2020).

Patients were included when they met the following inclusion criteria: patients aged >18 years, ability to express informed consent, and endoscopic findings of colonic diverticula. Exclusion criteria were as follows: inability to sign informed consent and to perform anamnesis collection, impossibility to perform multiple biopsies during colonoscopy (concomitant anticoagulant therapy and/or expired clinical conditions or concomitant conditions predisposing to high risk of bleeding), and the diagnosis of chronic inflammatory bowel disease (IBD) or other gastrointestinal clinical conditions (i.e., infectious, actinic, ischemic or drug-induced colitis) [11,12].

Patients whose colonoscopy showed endoscopic signs of interdiverticular mucosal inflammation (erythema, friability, and ulcerations) were suspected to have SCAD. Biopsies were taken from interdiverticular mucosa and the apparently normal adjacent mucosa as well as on the colon proximal to the diverticular area and rectum (at least 8 samples).

Histological analysis of biopsies was performed by an experienced gastrointestinal pathologist (E.P.). SCAD was defined by the presence of chronic inflammatory damage of interdiverticular mucosa (increased lymphoplasmacellular infiltrate, basal plasmocytosis and basal lymphoid aggregates, neutrophilic cryptitis, crypt abscesses, and crypt distortion) [13]. Inflammatory changes sparing proximal colon and rectum mucosa allowed differential diagnosis with other colonic conditions (i.e., IBD lesions, infective, ischemic colitis) [13,14].

Demographic and clinical features, including reasons for colonoscopy (i.e., CCS, positive fecal occult blood, hematochezia, rectal bleeding, anemia [15], abdominal pain, and alterations of bowel habits), were collected. In patients with suspected SCAD, the presence of gastrointestinal symptoms (hematochezia, rectal bleeding, alterations of bowel habits (constipation, diarrhea, mixed bowel habits), abdominal pain), medical history (including previous abdominal surgery and/or radiotherapy), pharmacological treatments (use of NSAIDs: occasional or frequent (at least once a week), antiplatelet agents, and anticoagulants), the type of intestinal preparation (high or low volume), or other drugs potentially inducing drug-induced colitis (i.e., proton pump inhibitors, antidiarrheal therapies, immunomodulators, antibiotics, antipsychotics, diuretics, selective serotonin reuptake inhibitors, tricyclic antidepressants, angiotensin-converting enzyme inhibitors, and statins) [11,12] were collected.

Colonoscopies were performed by expert endoscopists who reported the distribution and extension of the diverticula (sigmoid colon, left colon, right colon, and pancolonic), macroscopic signs of interdiverticular mucosal inflammation (erythema, friability and ulcerations), and the presence of other relevant colonic endoscopic findings.

Patients with colonic diverticulosis were compared with suspected SCAD patients based on demographic and clinical features, reasons for the colonoscopy, and location of the diverticula.

### Statistical Analysis

Data are expressed as a percentage (%) of the total or median (range) ± standard deviation (SD) for continuous variables. Univariate analyses were performed by the Mann–Whitney test (for continuous variables), and Fisher’s exact and chi-squared tests (for categorical variables) to identify differences between colonic diverticulosis and suspected SCAD. Two-tailed *p*-values < 0.05 were considered statistically significant. The statistical analysis was carried out using a dedicated software package (MedCalc Software, Mariakerke, Belgium, version 12.2).

## 3. Results

From 1 September 2019 to 28 February 2020, 1383 patients (711 females (51.4%), median age 65 ± 12.6 years (21.0–93.0)) underwent a colonoscopy (74.8% outpatients) at our tertiary university center. Diverticulosis was present in 367 patients (26.5%) (181 females (49.3%), median age 70 ± 10.6 years (38.0–93.0)). Compared to patients without diverticula (63 ± 15.4 years (21.0–91.0)), patients with diverticulosis were older (*p* < 0.0001), however no sex differences were found (females 49.3 vs. 51.4%, *p* = 0.482).

Among diverticulosis patients, 16 patients (4.4%) (9 females (56.3%), median age 60 ± 12.9 years (41.0–86.0)) presented macroscopic signs of inflammation of the interdiverticular mucosa and were identified as patients with suspected SCAD. Regarding the endoscopic signs of mucosal inflammation, all patients with suspected SCAD showed erythema of the interdiverticular mucosa, and one patient had fibrin erosion. No patient had a friable or ulcerated interdiverticular mucosa. The endoscopic findings of each patient with suspected SCAD together with demographic and clinical features (including medical history and concomitant treatment) are reported in Table 1. Half of the patients with suspected SCAD were asymptomatic, whereas the remaining patients experienced one or more gastrointestinal symptoms, including abdominal pain, hematochezia, and changes in bowel habits. Only one patient experienced rectal bleeding. Regarding the patient’s medical history, greater than half of the suspected SCAD patients (56.3%) did not report remarkable comorbidities, whereas less than one-fifth of the patients presented a history of oncological (18.7%) or cardiovascular disease (12.5%). One patient reported concomitant frequent use of NSAIDs for spondyloarthropathy, and another patient reported anticoagulant treatment (edoxaban) for atrial fibrillation. Almost all patients (93.8%) underwent high-volume intestinal preparation for colonoscopy.

The prevalence of diverticulosis in subjects undergoing colonoscopy for CCS was higher when compared to patients referred for symptoms and/or signs (51.3% vs. 13.2% *p* < 0.0001); this was probably attributable to the older age of patients with diverticulosis compared to patients without diverticula. Conversely, the prevalence of suspected SCAD was higher but not statistically significant among patients undergoing colonoscopy for CCS compared to patients referred for symptoms and/or signs (2% vs. 0.7% *p* = 0.05).

Compared to patients with diverticulosis, the age of suspected SCAD patients was significantly reduced at 60 years (41.0–86.0) vs. 70 years (38.0–93.0) (*p* = 0.047), but no sex differences were found (female 56.3 vs. 49.3% *p* = 0.619).

**Figure 1:**
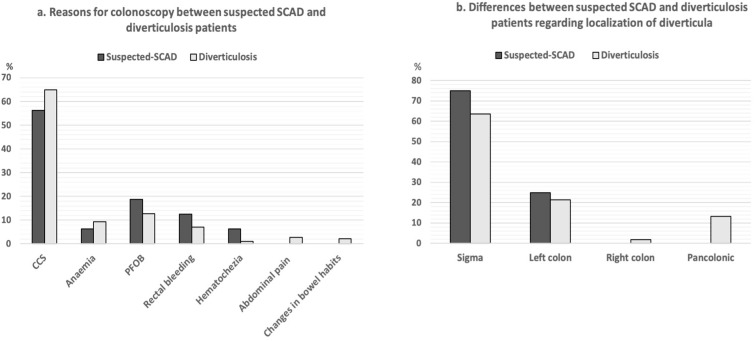
(**a**) Reasons for colonoscopy: comparison of patients with suspected SCAD and patients with colonic diverticulosis; (**b**) localization of diverticula: comparison between patients with suspected SCAD and patients with diverticulosis. SCAD: segmental colitis associated with colonic diverticulosis, CCS: colon cancer screening, PFOB: positive fecal occult blood.

No significant differences regarding reasons for colonoscopy between suspected SCAD and diverticulosis patients were identified (CCS 56.3 vs. 64.8% (*p* = 0.595); anemia 6.2 vs. 9.3% (*p* = 1.000); positive fecal occult blood 18.8 vs. 12.8% (*p* = 0.450); rectal bleeding 12.5 vs. 7.1% (*p* = 0.329); hematochezia 6.2 vs. 1.1% (*p* = 0.193); abdominal pain 0 vs. 2.7% (*p* = 1.000); changes in bowel habits 0 vs. 2.2% (*p* = 1.000)) (Figure 1a). Regarding the localization of diverticula, no significant differences were found between patients with suspected SCAD and patients with colonic diverticulosis, shown in the following: sigmoid (75.0% vs. 63.5%; *p* = 0.433); left colon (25.0% vs. 21.3%; *p* = 0.756); right colon (0% vs. 1.9%; *p* = 1.000); pancolonic (0% vs. 13.4%; *p* = 0.241) (Figure 1b).

### Histological Examination

**Figure 2:**
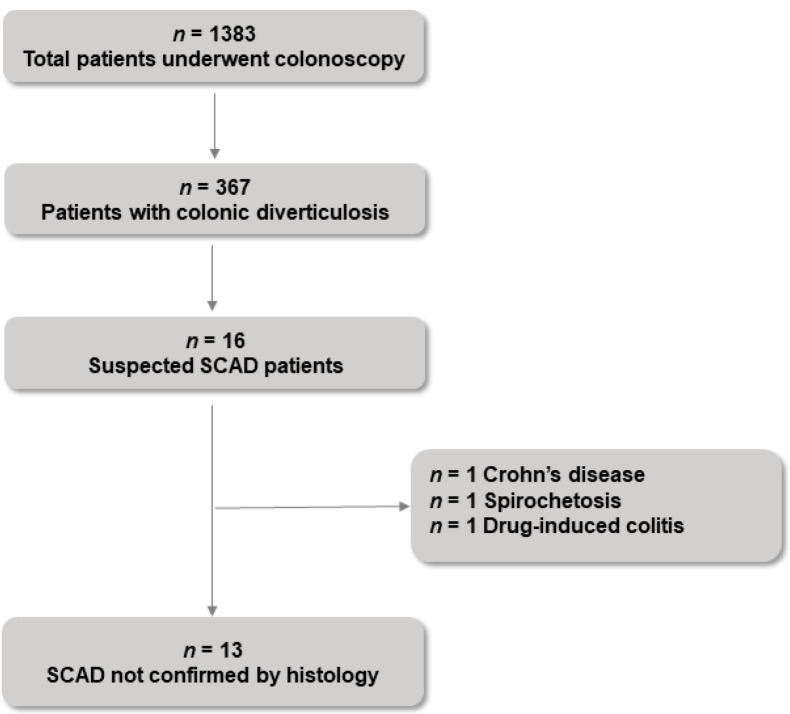
A flowchart of the study population. SCAD: segmental colitis associated with diverticulosis.

No patient with suspected SCAD was confirmed as having SCAD at histology. A flowchart of the study population is shown in Figure 2. One patient had a severe increase in lymphoplasmacytic infiltrate in the lamina propria, focal basal lymphoplasmacytosis, cryptic abscesses with mild alterations of crypt architecture in samples from both the sigma and rectum, which is compatible with a new diagnosis of chronic inflammatory bowel disease as Crohn’s disease. One patient presented a continuous basophilic border on the superficial epithelium of the sigmoid and rectum mucosa, which is compatible with intestinal spirochetosis. One patient using NSAIDs presented mild chronic inflammatory interstitial infiltrate (involving the right colon, interdiverticular mucosa, and rectum) associated with lymphoid aggregates of the lamina propria (only in the rectum), which is compatible with drug-induced colitis. The remaining patients with suspected SCAD showed edema and blood extravasations involving both the sigma and rectum.

## 4. Discussion

This observational study aims to evaluate the prevalence of SCAD in consecutive patients with endoscopic findings of diverticulosis of the colon. Although less than 5% of diverticulosis patients were identified as having suspected SCAD, none of these patients were confirmed by histological examination. In fact, the majority of patients had edema and blood extravasations in both the rectum and interdiverticular mucosa. In addition, one patient had a new diagnosis of Crohn’s disease, one patient presented lesions compatible with intestinal spirochetosis, and one presented with drug-induced colitis.

To the best of our knowledge, only a few studies describing SCAD prevalence are available, and the prevalence of this condition in diverticulosis is still being assessed and currently ranges from 1.9 to 11.4% [9,10]. Previous studies have shown large heterogeneity regarding both the biopsy sampling protocol (biopsies of the proximal colon are often lacking) and the histological criteria that ranged from nonspecific mild inflammation to IBD-like lesions. Recently, SCAD has also been recognized in patients with COVID-19 infection [16].

In a multicenter prospective study, Imperiali et al. found a prevalence of 1.9% in a total of 733 diverticulosis cases. However, the diagnosis of SCAD may be questionable since biopsies of the colon proximal to the interdiverticular area are lacking, the number of biopsies is not reported, and the histological examination of the interdiverticular mucosa showed only nonspecific inflammation [9].

Uncertain diagnostic criteria were similarly identified in a retrospective study by Koutroubakis et al., showing a 3.8% SCAD prevalence in patients with diverticulosis based mainly on endoscopic criteria. In fact, the histological features were predominately heterogeneous, and only 1% had histology compatible with SCAD. In addition, the biopsy sampling protocol was unclear and did not include the colon proximal to the interdiverticular mucosa [17].

In another study by Gore et al., a prevalence of 1.4% was reported; however, the authors included patients with signs of inflammation in the crescentic mucosal folds, even without diverticulosis (only 82% of patients had diverticula), and no biopsies of the colon proximal to the inflammation were performed [18].

A more recent study conducted by Tursi et al. reported a prevalence of SCAD of 11.4% among 807 patients with colonic diverticulosis, which is the highest proportion among available studies [10]. The authors recognized four different endoscopic patterns of SCAD (all including macroscopic signs of inflammation in the interdiverticular mucosa, with spared proximal and rectal colon), among which the most frequent pattern was “crescentic fold disease”, which involves swollen red patches of the colonic mucosa, without hemorrhage or ulceration confined to the crescentic mucosal folds. Although the endoscopic scenario was similar to the majority of this study’s suspected SCAD cases and the biopsy sampling protocol overlapped with our study, the prevalence of SCAD was greater than in our study (11.4% vs. 0%). It is likely that different histological SCAD criteria (we excluded mild mucosal inflammation), different study designs (multicenter study involving several histopathologist vs. monocenter study with one expert histopathologist), and different lengths of the study duration (45 vs. 6 months) might explain this discrepancy. We are aware that the majority of our suspected SCAD patients presented mild endoscopic damage, i.e., less-specific SCAD lesions, but by adopting a more inclusive approach, we endeavored not to miss the description of possible inflammatory lesions. To achieve a correct differential diagnosis, we carefully collected the patient’s medical history and the pharmacological treatment of each patient; however, it should be noted that some mild signs of inflammation might be ascribed to colon cleansing. Recently, an endoscopic score (Diverticular Inflammation and Complication Assessment (DICA)) has been introduced to classify diverticular disease [19]. This score has been proposed to identify endoscopic findings predictive of diverticular disease outcome and includes SCAD as one of the items reported in the classification. Regardless, in cases of SCAD suspicion, histological examination is always mandatory to confirm the diagnosis and eventually address the treatment.

In the present study, if assessments were based mainly on endoscopic findings, it would have presented similar percentages of SCAD diagnoses compared to previously studied data (5.4%); nevertheless, even in the absence of shared diagnostic criteria, the gold standard for SCAD diagnosis is represented by histological assessment. The authors are aware that the present study is based on a histologic definition that may appear too rigid. However, no consensus regarding the histologic criteria is available, and various heterogeneous criteria were considered in clinical practice [2]. Thus, considering the importance of histologic assessment, further studies are needed to achieve shared diagnostic SCAD criteria.

A limitation of the present study is that the enrollment period was prematurely interrupted due to the COVID-19 health emergency, due to which routine endoscopic activity was suspended.

However, the present study presents more than one strength. We conducted an observational monocenter study on a prospective cohort of patients following a well-designed diagnostic protocol with an expert GI pathologist.

Although less than 5% of diverticulosis patients were identified as suspected SCAD at endoscopy, none of them were confirmed at the histology examination.

## 5. Conclusions

In conclusion, this observational study suggests that SCAD diagnosis is a challenge in clinical practice due to the heterogeneity of endoscopic findings and lack of stated histological criteria.

## Figures and Tables

**Table 1:** Demographic, clinical features and endoscopic findings in patients with suspected SCAD.

Patient	Age	Sex	BMI kg/m^2^	GI Symptoms	Medical History	Concomitant Treatment	Intestinal Preparation	Endoscopic Findings of Interdiverticular Mucosa	Histological Examination	Other Findings
**1**	59	F	22.9	Abdominal pain, constipation	Breast cancer	-	HV	Erythema	^1^ SCAD not confirmed	Micropolyp of sigma (hyperplastic)
**2**	57	F	26.8	None	-	-	HV	Erythema	^1^ SCAD not confirmed	Three micropolyps of left and right colon (tubular adenoma, low grade dysplasia)
**3**	55	M	24	None	-	-	HV	Erythema	^1^ SCAD not confirmed	Micropolyp of left colon (tubular adenoma, low grade dysplasia)
**4**	79	F	25	Hematochezia	Previous diverticulitis	-	HV	Erythema	Crohn’s disease	Two micropolyps of right colon (hyperplastic)
**5**	86	M	23.3	None	-	-	HV	Erythema	^1^ SCAD not confirmed	Pedunculated polyp of sigma (tubular adenoma, low grade dysplasia), sessile polyp of transverse colon (hyperplastic)
**6**	41	M	33.9	None	Previous diverticulitis	-	HV	Erythema	^1^ SCAD not confirmed	-
**7**	76	F	30.3	Hematochezia	Breast cancer	-	HV	Erosions with fibrin, erythema	^1^ SCAD not confirmed	Moderate chronic inflammatory infiltrate and edema, in sigma and rectum, with spared right colon
**8**	46	M	25.8	Abdominal pain	-	-	HV	Erythema	Spirochetosis	-
**9**	52	M	22	None	-	-	HV	Erythema	^1^ SCAD not confirmed	-
**10**	52	F	32	Abdominal pain, constipation	Breast cancer, spondyloarthropathy	NSAIDs	HV	Erythema	Drug-induced colitis	Micropolyp of right colon (hyperplastic)
**11**	72	M	24.6	None	-	-	HV	Erythema	^1^ SCAD not confirmed	Sessile polyp of right colon (villous-tubular adenoma, low grade dysplasia)
**12**	54	F	24.3	None	-	-	HV	Erythema	^1^ SCAD not confirmed	-
**13**	72	F	23.8	Abdominal pain, hematochezia, diarrhea	Cardiovascular disease	-	HV	Erythema	^1^ SCAD not confirmed	Pedunculated polyp of sigma (villous-tubular adenoma, low grade dysplasia
**14**	61	F	24	None	-	-	LV	Erythema	^1^ SCAD not confirmed	-
**15**	73	F	26.7	Abdominal pain	-	-	HV	Erythema	^1^ SCAD not confirmed	Micropolyp of left colon (hyperplastic)
**16**	72	M	24.3	Rectal bleeding	Cardiovascular disease, atrial fibrillation	Anticoagulant	HV	Erythema	^1^ SCAD not confirmed	Sessile polyp of right colon (histology not available)

^1^ Edema and blood extravasations involving both the sigma and rectum. BMI: body mass index, F: female, GI: gastrointestinal, HV: high volume, LV: low volume, M: male, SCAD: segmental colitis associated with diverticulosis, NSAIDs: nonsteroidal anti-inflammatory drugs.

## Data Availability

Data sharing not applicable.
